# Exercise-induced left bundle branch block and subsequent mechanical left ventricular dyssynchrony -resolved with pharmacological therapy

**DOI:** 10.1186/1476-7120-9-4

**Published:** 2011-02-07

**Authors:** Hidekazu Tanaka, Mana Hiraishi, Tatsuya Miyoshi, Takayuki Tsuji, Akihiro Kaneko, Keiko Ryo, Kohei Yamawaki, Yuko Fukuda, Kazuko Norisada, Kazuhiro Tatsumi, Kensuke Matsumoto, Hiroya Kawai, Ken-ichi Hirata

**Affiliations:** 1 Division of Cardiovascular Medicine, Department of Internal Medicine, Kobe University Graduate School of Medicine, Kobe, Japan

## Abstract

A 53-year-old man with depressed ejection fraction (EF) of 35% and QRS width of 88 ms at rest was admitted to our institution with a complaint of exertional chest discomfort and dyspnea. During treadmill exercise, left bundle-branch block (LBBB) with a QRS width of 152 ms occurred at a heart rate of 100 bpm. During LBBB, the patient showed significant mechanical dyssynchrony as evidenced by a two-dimensional speckle tracking radial strain of 260 ms (≥130 ms), defined as the time difference between anterior-septum and posterior wall. Five-month after carvedilol and candesartan administration, EF had improved to 49% and LBBB did not occur until a heart rate of 126 bpm was attained during treadmill exercise. It appears that pharmacological therapy may be useful for patients with heart failure and exercise-induced LBBB.

## Background

The occurrence of left bundle branch block (LBBB) during exercise testing is a relatively rare occurrence. In fact, only approximately 0.5%-1.1% of all patients who undergo exercise testing develop a transient LBBB during exercise[1,2]. While the exact causative mechanism for exercise-induced LBBB remains unclear, it may reflect underlying myocardial dysfunction, structural heart disease, or compromised coronary circulation. LBBB is known to impair the mechanical function of the left ventricle, and previous studies have shown that LBBB is associated with increased mortality, while the relative risk associated with the presence of LBBB in these studies varied roughly between 1.5 and 2.0, even after adjustment for covariates[3-6].

LBBB is characterized by early septal radial inward thickening, followed by late posterior inward thickening, which then results in a significant LV dyssynchrony. LV dyssynchrony has emerged as an important mechanism contributing to progression of heart failure and ventricular remodeling, and appears to play a major pathophysiologic role in heart failure. LV dyssynchrony affects LV diastolic function, right ventricular and left atrial function as well as LV systolic function. This report concerns a 53-year-old man with exercised-induced LBBB and subsequent mechanical LV dyssynchrony, which was resolved with pharmacological therapy.

## Case presentation

**Figure 1:**
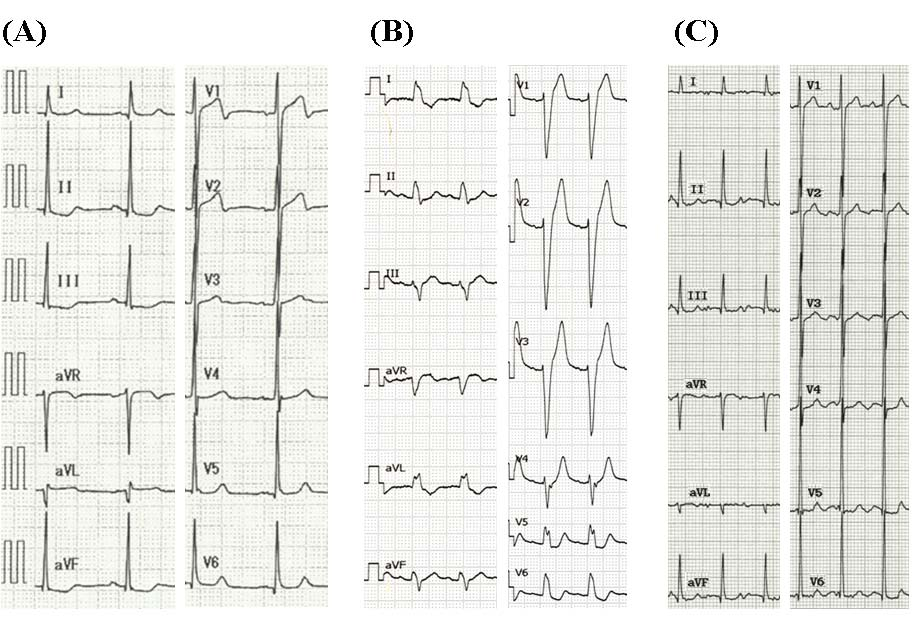
**12-lead electrocardiogram**. **(A)** 12-lead electrocardiogram taken at rest shows a QRS width of 88 ms. **(B) **12-lead electrocardiogram after treadmill exercise test indicates a left bundle branch block with a QRS width of 152 ms at a heart rate of 100 bpm. **(C) **12-lead electrocardiogram following the treadmill exercise test 5-month after pharmacological therapy does not show any a left bundle branch block at a heart rate of 126 bpm.

**Figure 2:**
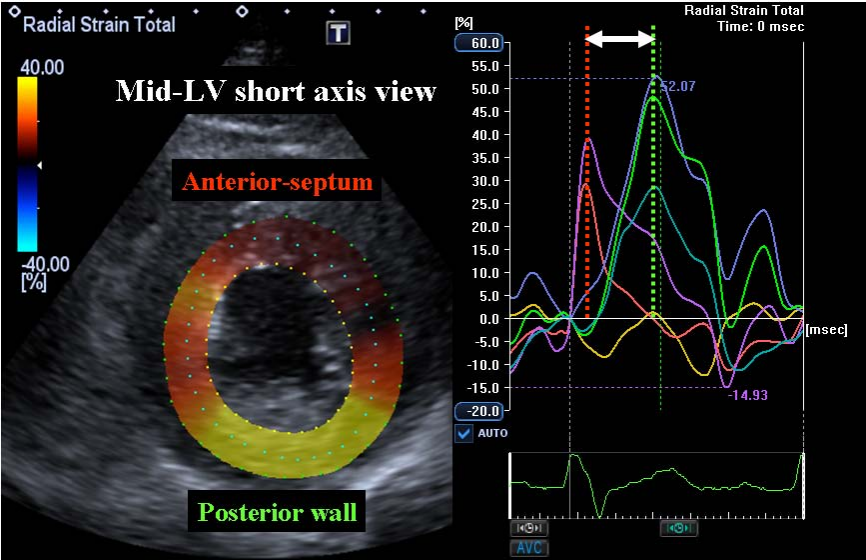
**Speckle tracking radial time strain curves derived from a mid-left ventricular short-axis image with left bundle branch block after treadmill exercise test**. Significant mechanical dyssynchrony is indicate by the time difference (white arrow) of 260 ms between time-to peak strain in the anterior septum (red line) and to posterior wall peak strain (green line).

**Figure 3:**
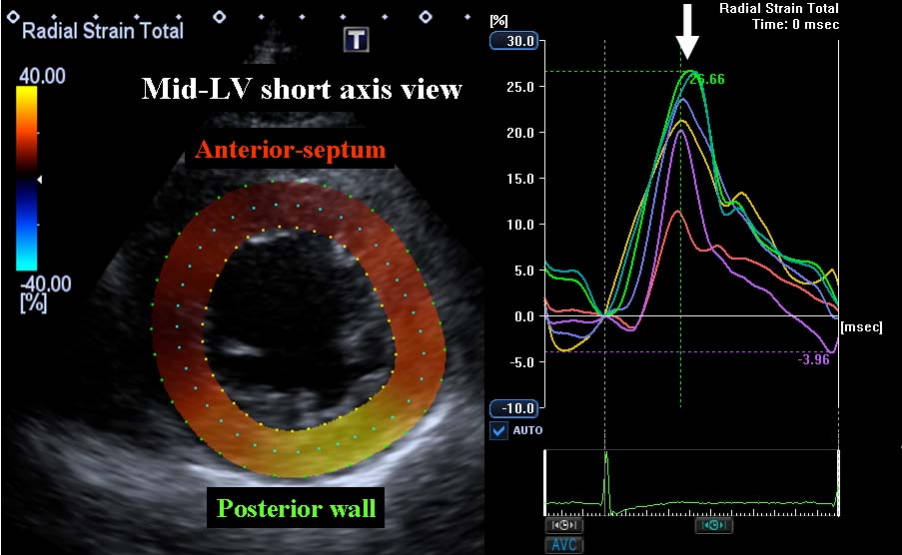
**Speckle tracking radial time strain curves derived from a mid-left ventricular short-axis image after disappearance of the left bundle branch block**. Speckle tracking radial dyssynchrony of 45 ms was not significant.

A 53-year-old man was admitted to our institution with a complaint of exertional chest discomfort and dyspnea. Physical examination and chest radiography findings were normal. The 12-lead electrocardiogram taken at rest revealed normal sinus rhythm with a QRS width of 88 ms (Figure 1A). Echocardiographic examination was performed to assess LV function (Aplio Artida, Toshiba Medical Systems Corporation, Tochigi, Japan). The LV ejection fraction (EF) calculated with biplane Simpson's rule was 35%, LV end-diastolic diameter was 55 mm and left ventricular end-systolic diameter was 40 mm (Table 1, Additional file 1: Video 1). No abnormalities were found in the mitral and aortic valve. Because of the patient's history of exertional chest discomfort and dyspnea, treadmill stress echocardiography according to the Bruce protocol was administrated. Before exercise, heart rate was 60 bpm and blood pressure was 136/80 mmHg, and 4 minutes after starting the exercise, LBBB with a QRS width of 152 ms occurred at a heart rate of 100 bpm (Figure 1B), but the patient did not develop chest pain or any other symptoms during stress testing. During LBBB, the patient showed significant mechanical dyssynchrony as evidenced by a speckle tracking radial strain of 260 ms (≥130 ms)[7,8], defined as a time difference between anterior-septum and posterior wall (Figure 2 Additional file 2: Video 2). When echocardiographic dyssynchrony analysis was repeated with the patient at rest after LBBB had disappeared, the dyssynchrony of 45 ms, again detected by speckle tracking radial strain, was not significant (Figure 3).

**Table 1:** Resting echocardiographic characteristics in the patient

	Baseline	5-month after pharmacological therapy
Left ventricular end-diastolic diameter (mm)	55	46
Left ventricular end-systolic diameter (mm)	37	33
Left atrial diameter (mm)	33	31
Thickness of interventricular septum (mm)	13	12
Thickness of posterior wall (mm)	12	12

Coronary angiography did not reveal significant stenosis, and LVEF was 35%, pulmonary capillary wedge pressure 11 mmHg, pulmonary artery pressure 23/6 mmHg, and cardiac index 3.01 l/min/m^2^. Histological examination of the excised right ventricle showed none of the significant features of cardiomyopathy.

The patient was treated with final daily dosages of 20 mg carvedilol and 4 mg candesartan for depressed LV systolic function. Five-month after administration of the medication, LVEF had improved to 49% as calculated with biplane Simpson's rule (Additional file 3, Video 3). The treadmill exercise test according to the Bruce protocol was also performed. LBBB did not occur until a heart rate of 126 bpm had been reached (Figure 1C).

## Discussion

The case reported here concerns a patient with exercise-induced LBBB and subsequent significant mechanical LV dyssynchrony assessed by two-dimensional speckle tracking radial strain. The most important aspect of this case is that exercise-induced LBBB and LV function were resolved with pharmacological therapy. To the best of our knowledge, this is the first report in the literature of such a case.

The occurrence of LBBB during exercise testing is a relatively rare finding. In fact, only approximately 0.5%-1.1% of all patients who undergo exercise testing develop a transient LBBB during exercise[1,2]. The precise causative mechanism for exercise-induced LBBB remains unclear, but it may be a reflection of underlying myocardial dysfunction, structural heart disease, or compromised coronary circulation. It was first suggested that it is caused most often by occlusive coronary artery disease, but several authors have reported patients who developed exercise-induced LBBB even though coronary angiography findings appeared normal. Furthermore, Loubeyre et al suggested the presence of microcirculatory ischemia undetectable by coronary angiography as a possible mechanism for exercise-induced LBBB [9]. The prognostic significance of exercise-induced LBBB is also poorly understood. The general consensus in the literature is that the prognosis of exercise-induced LBBB is good if there is no underlying structural heart disease[10,11]. On the other hand, Grady et al used a large patient series to demonstrate that exercise-induced LBBB can be an independent predictor of major cardiovascular morbidity and mortality[1]. It has also been suggested that another prognostic factor is the heart rate at which exercise-induced LBBB occurs. That is, the onset of exercise-induced LBBB at a heart rate of 120-125 bpm or lower correlated strongly with the presence of occlusive coronary artery disease, whereas patients who develop exercise-induced LBBB at a heart rate of 120-125 bpm or higher show normal-appearing coronary arteriograms and have a better prognosis[10]. In the case presented in our report, the heart rate at onset of exercise-induced LBBB was recorded at 100 bpm but the patient did not develop any symptoms suggestive of angina pectoris. While the precise reason for the effect of pharmacological therapy on exercise-induced LBBB remains unknown, such therapy may simply shift the heart rate at which LBBB ensues because the onset of LBBB is heart rate related.

LV dyssynchrony impairs LV diastolic, right ventricular and left atrial function as well as LV ejection efficiency. LV dyssynchrony has therefore emerged as an important mechanisms contributing to the progression of heart failure and ventricular remodeling, and appears to play a major pathophysiologic role in heart failure. Since roughly one-third of heart failure patients with a wide QRS width do not show significant LV dyssynchrony[8,12], the quantification of LV dyssynchrony by means of echocardiography could be important for assessment of heart failure patients. In our case, the patient's symptom appeared during treadmill exercise, and LBBB with LV dyssynchrony during the activity impaired his LV function (Table 2). Thus, exercise-induced LV dyssynchrony might constitute a warning for this patient. Because LBBB is characterized by early septal radial inward thickening, followed by late posterior inward thickening, we focused on radial thickening as a major vector of LV contraction and short-axis dynamics as important markers for the assessment of LV dyssynchrony. LV dyssynchrony has emerged as an important mechanisms contributing to the progression of heart failure and ventricular remodeling, and appears to play a major pathophysiologic role in heart failure. According to previous studies involving radial strain detected by speckle tracking, baseline speckle tracking radial dyssynchrony, defined as a time difference in peak septal-to-posterior wall strain ≥ 130 ms, predicted chronic response to CRT[7,8]. Because this phenomenon was observed only in a case with a typical LV dyssynchrony pattern, the presence of this phenomenon needs to be confirmed in a case without LV dyssynchrony.

**Table 2:** Resting and peak stress clinical and echocardiographic characteristics at baseline and follow-up in the patient

	Baseline		5-month after pharmacological therapy	
	Rest	Peak stress	Rest	Peak stress
Clinical parameters				
Heart rate (bpm)	60	138	54	135
Systolic blood pressure (mmHg)	136	184	118	176
Diastolic blood pressure (mmHg)	64	106	58	102
Double product (mmHg/min)	8160	25392	6372	23760
Echocardiographic parameters				
LV end-diastolic volume (ml)	108	109	87	88
LV end-systolic volume (ml)	70	76	44	40
LV ejection fraction (%)	35	30	49	54
Mitral inflow pattern	impaired LV relaxation	impaired LV relaxation	normal	normal
E/E'	18.9	19.3	11.4	10.9
Radial dyssynchrony by speckle tracking strain (ms)	45	260	42	48

E, early diastolic wave velocity; E', early diastolic mitral annular velocity; LV, left ventricular

## Conclusions

Exercise-induced LBBB with significant mechanical LV dyssynchrony may constitute an important prognostic finding for patients with heart failure. Moreover, pharmacological therapy using drugs such as carvedilol and candesartan may be useful for the treatment of exercise-induced LBBB. Because ours was an isolated case, further clinical studies are required to validate this finding.

## List of abbreviations

EF: ejection fraction; LBBB: left bundle-branch block; LV: left ventricular.

## Competing interests

The authors declare that they have no competing interests.

## Consent

Written informed consent was obtained from the patient for publication of this case report and any accompanying images. A copy of the written consent is available for review by the Editor-in-Chief of this journal.

## Authors' contributions

HT designed the study, carried out subject recruitment, performed echocardiography, analysed the data, and wrote the manuscript. MH, TM, TT, AK, KR, KY, YF, KN, KT, KM, HK, and KH assisted recruitment and manuscript revision. All authors read and approved the final manuscript.

## Supplementary Material

Additional file 1 **Apical 4-chamber view at baseline, showing left ventricular ejection fraction of 35% determined with biplane Simpson's rule**. Click here for file

Additional file 2 **Apical 4-chamber view with left bundle branch block during treadmill exercise, showing left ventricular mechanical dyssynchrony**. Click here for file

Additional file 3 **Apical 4-chamber view 5-month after pharmacological therapy, showing left ventricular ejection fraction of 49% determined with biplane Simpson's rule**. Click here for file
